# In a Pinch: An Unusual Case of Zoon’s Vaginitis, a Plasma Cell Disorder

**DOI:** 10.7759/cureus.43260

**Published:** 2023-08-10

**Authors:** Kristina Karlson, Dane Fishburn, Katerina Shvartsman, Sara Elling, Andrea Snitchler, Elaine Keung

**Affiliations:** 1 Gynecologic Surgery and Obstetrics, Walter Reed National Military Medical Center, Bethesda, USA; 2 Gynecologic Surgery and Obstetrics, Uniformed Services University of the Health Sciences, Bethesda, USA; 3 Pathology, Walter Reed National Military Medical Center, Bethesda, USA

**Keywords:** vaginal steroids, vaginitis, vulvitis, plasma cell, zoon’s

## Abstract

Plasma cell, or Zoon’s, vulvitis (PCV) is a rare inflammatory disorder of the female genital tract. Clinically, it is characterized by erythematous mucosal lesions associated with burning, pruritus, and dyspareunia. Histologically, it is characterized by the thinning of the epithelium with the infiltration of plasma cells in the underlying dermis. There are few case reports describing predominantly vaginal symptoms.

Our patient is a 53-year-old postmenopausal female presenting for the gynecologic evaluation of a vaginal pinching sensation and vulvar irritation for three months. On examination, vaginal mucosa was notable for erythematous macules and papules with focal tenderness. Initial evaluation was significant for bacterial vaginosis. This was treated, but it did not improve the patient’s presenting symptoms. Our preliminary working diagnosis was vulvovaginal atrophy. Biopsies showed plasmacytosis mucosae consistent with Zoon’s vaginitis. The patient was treated with external clobetasol ointment and hydrocortisone 25 mg vaginal suppositories with improvement in symptoms.

Female genital tract lesions engender a range of differential diagnoses, including infectious, immunologic, and malignant causes. In this patient, our initial working diagnosis of genitourinary syndrome of menopause suggested that local hormonal treatment was indicated. However, histological diagnosis directed the use of steroid treatment, ultimately improving the patient’s symptoms.

## Introduction

Plasma cell, or Zoon’s, vulvitis (PCV) is a rare but known idiopathic inflammatory disorder of the female genital tract first described in the 1950s [1]. Typically, this disorder is described as affecting the vulva. However, there are several case reports and small case series involving the vagina and cervix, with the first case described in 1999 [1-4]. The prevalence of vaginal plasma cell disorder is unknown and likely underreported as typical symptoms overlap with several more common vulvovaginal disorders such as lichen sclerosus, lichen planus, infectious vaginitis, pemphigus, pemphigoid, contact dermatitis, and genitourinary syndrome of menopause [5-7]. Common vulvar symptoms include burning, itching, and dyspareunia, although some patients may be asymptomatic [1-3,8]. In the vagina, abnormal discharge, including a yellow or leukorrhea-type discharge, has been described [1,2].

The most described examination finding is well-circumscribed, erythematous, and glistening patches with a faint red-orange hue. Histologically, findings consist of thinned epithelium with the infiltration of greater than 50% plasma cells in the underlying dermis [1,4,5]. It is recognized that PCV is classically misdiagnosed in cases of vaginal predominant disease [2]. Currently, there are only four reported cases of plasma cell vaginitis [1-4]. Our case demonstrates the importance of vaginal biopsy in timely diagnosis and treatment.

## Case presentation

A 53-year-old postmenopausal female was referred to the gynecology clinic for the evaluation of vulvar irritation and vaginal pinching sensation for the past three months. The pinching sensation occurred with movement, especially when bending over. The patient denied vaginal discharge, vulvovaginal itching, dysuria, and pain with intercourse. She denied the use of new detergents or baby wipes. When symptoms first began, she was evaluated by her primary care provider, who recommended that the patient wear looser-fitting clothing; however, this modification did not improve her symptoms. The patient’s gynecologic history is notable for two pap smears showing atypical squamous cells of undetermined significance (2007 and 2009), pelvic inflammatory disease in the setting of trichomonas (2013), and uterine artery embolization for leiomyomas (2014). Her medical history is notable for hypertension, gastroesophageal reflux disease, depression, migraines, seasonal allergies, reactive airway disease, and surgical excision of breast fibroadenoma. Her medications include hydrochlorothiazide, lisinopril, omeprazole, and fexofenadine. The patient has no known drug allergies.

On examination, the vulva appeared normal. The vaginal mucosa was notable for focal tenderness on opposing side walls with multiple 2-3 mm, flat, glossy, erythematous macules and papules covering an approximately 1 cm area located 3 cm cephalad to the hymen. Clinical photos were not taken at this time. The patient underwent testing for bacterial vaginosis, yeast, trichomonas, gonorrhea, chlamydia, HIV, hepatitis, and syphilis. Test results were positive for bacterial vaginosis, for which the patient received treatment with metronidazole 500 mg twice a day for seven days. Biopsy was not performed at this time due to the profoundly erythematous appearance of the papules; instead, an MRI was obtained to evaluate for a possible underlying vascular lesion. The MRI did not show any vaginal abnormalities. Based on the patient’s history and examination, our working diagnosis was genitourinary syndrome of menopause; however, we planned to re-evaluate the patient after she completed her treatment for bacterial vaginosis and perform a biopsy to complete a comprehensive evaluation prior to initiating estrogen therapy.

**Figure 1:**
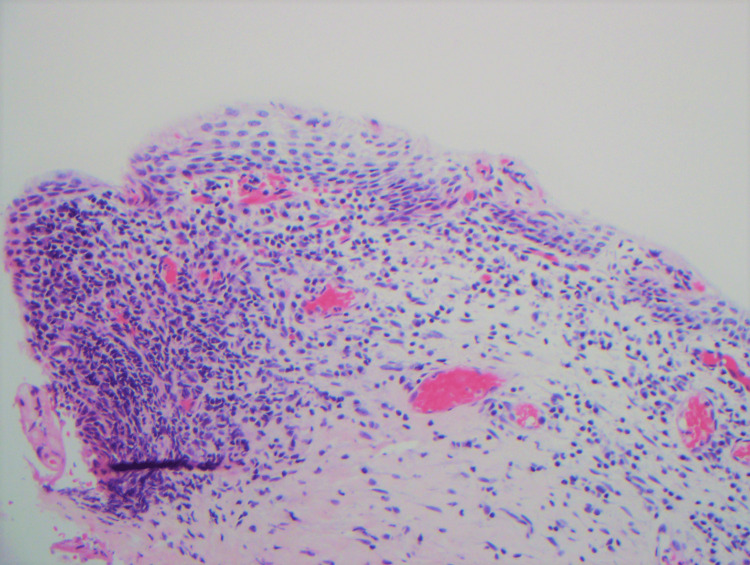
Hematoxylin and eosin (20×) The image shows epidermal atrophy with a band-like infiltrate involving the upper dermis and a prominent dilated blood vessel

**Figure 2:**
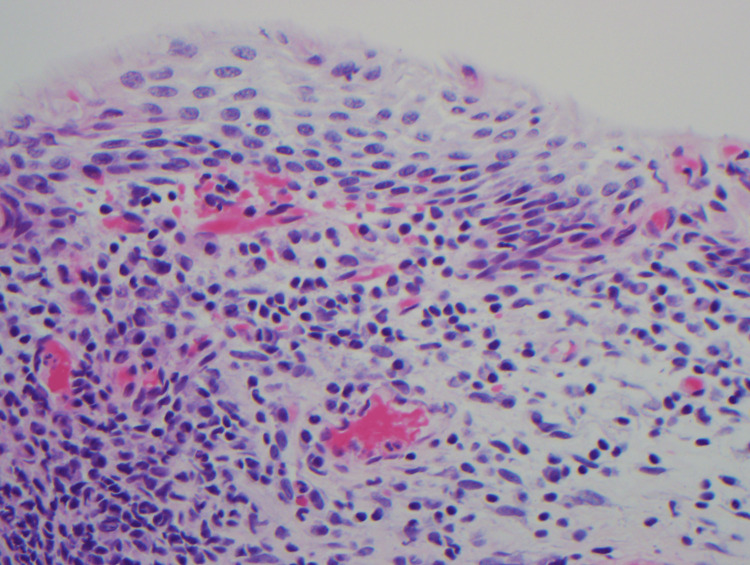
Hematoxylin and eosin (40×) The epidermis exhibits spongiosis and atrophy overlying a dermis with a band-like inflammation composed of polytypic plasma cells, lymphocytes, and neutrophils. Dilated superficial vessels with extravasated erythrocytes are also present

Two weeks after the treatment of bacterial vaginosis, two biopsies were performed using Tischler forceps, one from the right and one from the left vaginal sidewall. Repeat bacterial vaginosis testing was not performed. The biopsies showed plasmacytosis mucosae consistent with Zoon’s vaginitis (Figure 1 and Figure 2). Histopathology exhibited mild epidermal spongiosis with atrophic changes overlying a band-like superficial dermal infiltrate composed of plasma cells, lymphocytes, and scattered neutrophils. Dilated superficial vessels with extravasated erythrocytes were also present. Additional stains were performed and revealed negative fungal and treponemal special stains. Kappa and lambda in situ hybridization (ISH) showed a polyclonal plasma cell population.

Upon reviewing the diagnosis with the patient, she reported the development of new-onset vulvar pruritus since her initial presentation. The patient was prescribed clobetasol 0.05% ointment to apply to the affected areas on the vulva nightly for two weeks and then twice per week thereafter, along with hydrocortisone 25 mg suppositories inserted vaginally twice daily for six weeks. She reported significant improvement in her symptoms on this regimen and did not require topical estrogen therapy. At one-year follow-up, the patient reported using hydrocortisone suppositories on one occasion with good response, and she was currently asymptomatic.

Due to the unique nature of the case, the patient consented to the presentation and publication of her case according to the Department of Defense policies and requirements.

## Discussion

Female genital tract lesions engender a range of causes including infectious, immunologic, and malignant causes. The differential diagnosis for patients presenting with vulvovaginal erythema or pruritus may include lichen sclerosus, lichen planus, psoriasis, lichen simplex chronicus, infectious vaginitis (*Gardnerella vaginalis*, candidal vaginitis, and *Trichomonas* vaginitis) or desquamative inflammatory vaginitis, contact dermatitis, pemphigus or pemphigoid, lupus erythematosus, drug reactions, genitourinary syndrome of menopause, extramammary Paget’s disease, and neoplasms [5,6,9-11].

In 2021, three systematic reviews were published on plasma cell vulvitis [12-14]. In these reviews, the average age of patients was between 52 and 55, with an approximately one-to-two-year delay in diagnosis. The most common symptoms reported in plasma cell disorders of the female genital tract were burning, pruritus, and dyspareunia. In cases specifically involving the vagina, patients often presented with copious discharge, vaginal bleeding, and pain. These symptoms overlap widely with more common conditions previously mentioned.

Additionally, several cases of vaginal plasma cell disorder reported concurrent infections that required treatment and were potential distractors or causes of delay in diagnosis until a biopsy was considered, as in our patient with bacterial vaginosis [1,2,8,9,15]. There is no available evidence to suggest that persistent bacterial vaginosis may result in plasma cell infiltrate if not adequately treated, and thus, repeat testing was not performed. While initial targeted management may appear as the best treatment for vaginitis, patients with severe, chronic, and unresponsive symptoms should undergo timely tissue diagnosis to guide appropriate therapy.

Histopathology plays a pivotal role in the correct diagnosis of PCV, allowing for appropriately directed treatment to gain clinical improvement. A plasma cell inflammatory infiltrate is the most common finding, which was consistent with our results [11,16]. Additionally, histopathology can aid in ruling out other disorders. For example, contact/allergic dermatitis and drug eruptions were considered in the differential but were less favored due to clinical history and the lack of eosinophils. The deep dermis was unremarkable, and the basal layer of the epidermis was intact without lichenoid or psoriasiform inflammatory changes without lymphocyte predominance making lichen planus, lichen sclerosus et atrophicus, variants of lupus erythematosus, and psoriasis unlikely. No spirochetes were present on staining, and serum testing for syphilis was also negative [16]. The presence of polyclonal plasma cells made the diagnosis of plasma cell vaginitis most likely.

As far as the management of PCV, treatment has not been standardized and even less so in cases isolated to the vagina. Treatment may include topical, oral, intralesional, procedural, and surgical options. Regarding recommended therapy, experts have varying opinions and success rates. Biopsy is emphasized as a necessity for diagnosis, with the most common treatment using varying regimens of topical corticosteroids (TCS) and tacrolimus [12-14,17].

In Sattler et al.’s review (53 publications), the most common TCS used was clobetasol and hydrocortisone. Other topical agents included immunomodulation with tacrolimus and imiquimod. Combination topical regimens were also trialed, including betamethasone with fusidic acid and clobetasol with tacrolimus. Systemic treatments were less common and included antibiotics, steroids, hormones, antivirals, antifungals, retinoids, and interferons. Procedural treatments included surgical excision, laser therapy, fulguration, and plate-rich plasma. Overall, this review supports the need for prospective studies on the characterization of the disorder and treatment guidelines [12].

In Yun and Veysey’s review (45 publications), similar treatment regimens were identified as the previous review; they noted that treatment with estrogen or antimicrobials before diagnosis did not resolve symptoms and therefore is not recommended as monotherapy. These treatments are often offered in the setting of concomitant diagnoses such as atrophic vaginitis, bacterial vaginosis, candidiasis, and sexually transmitted infections. Notably, studies were found to lack validated outcome measures, making it difficult to make strong recommendations for specific treatment modalities. Overall, this review supports the need for standardized outcome measures and recommends randomized clinical trials for therapeutic recommendations [13].

Lastly, Krapf et al.’s review (39 publications) notably included four cases of vaginal plasma cell disorder and recognized concomitant autoimmune disorders (5%) and sexually transmitted infections (6%). Similarly, the most common treatment modalities for PCV included TCS, tacrolimus, and imiquimod, with 88% of patients achieving symptom resolution. In the four cases with vaginal disease, treatments were directed intravaginally (creams and suppositories) with fusidic acid, corticosteroids, estrogen, and clindamycin versus surgical excision. Overall, this review recommends more definitive diagnosis criteria for PCV, including a thorough internal pelvic assessment in patients with existing vulvar predominant PCV and an evaluation of treatment efficacy [14].

Based on the review of the current literature, we chose a regimen of topical clobetasol 0.05% ointment applied to the affected areas on the vulva nightly for two weeks, followed by maintenance therapy twice a week along with hydrocortisone 25 mg suppositories inserted vaginally twice a day for six weeks. Our patient noted marked improvement on this regimen through 12 months. Further treatment and maintenance therapy should be guided by the patient’s symptoms and examination, as there are no prospective studies evaluating treatment in the setting of the chronic nature of the disorder. Yun and Veysey’s review noted recurrence in symptoms weeks to years after initial treatment, and recurrence in symptoms was least noted with topical corticosteroids. Unfortunately, the duration of initial therapy and whether recurrence occurred while on treatment are unknown [13].

## Conclusions

Patients who present with vulvovaginal complaints require a thorough history and physical examination with a low threshold for histological diagnosis and other diagnostic testing. Empiric treatment without comprehensive evaluation may result in missed diagnoses. Missed diagnoses may result in delays in treatment and potential long-term complications.

While vaginal predominant plasma cell disorder is an extremely rare form of PCV, with only four cases between case reports and case series to extract clinical data, treatment with topical corticosteroids proved a reliable, safe, and effective treatment for our patient. Directed therapy was made possible by collecting biopsies for histological evaluation. We recommend a low threshold for biopsy and initiating treatment with topical corticosteroids in patients without an identifiable infectious or inflammatory disorder of the vulva and vagina.
